# Spontaneous closure of degenerative lamellar macular hole with epiretinal membrane proliferation

**DOI:** 10.1186/s40942-021-00339-z

**Published:** 2021-10-26

**Authors:** Rony C. Preti, Leandro C. Zacharias, Leonardo P. Cunha, Mario L. R. Monteiro, David Sarraf

**Affiliations:** 1 Division of Ophthalmology, University of São Paulo Medical School, São Paulo, SP Brazil; 2 Department of Ophtalmology, School of Medicine, Federal University of Juiz de Fora, Juiz de Fora, Minas Gerais, Brazil; 3 David Geffen School of Medicine at UCLA, Stein Eye Institute, Los Angeles, CA USA; 4 Greater Los Angeles Veterans Affairs Healthcare Center, Los Angeles, CA USA

**Keywords:** Lamellar macular hole, Epiretinal proliferation, Epiretinal membrane, Lamellar hole–associated epiretinal proliferation, Spectral domain optical coherence tomography

## Abstract

**Background:**

To describe the spontaneous closure of a degenerative lamellar macular hole with epiretinal proliferation (LHEP) as documented with tracked spectral domain optical coherence tomography (SD-OCT).

**Case presentation:**

A 54-years-old diabetic female patient presented with progressive vision loss in the left eye. SD-OCT illustrated LHEP associated with cystic fluid in the outer nuclear layer. Sequentially tracked SD-OCT showed progressive closure of the degenerative lamellar macular hole and resolution of the CME over almost 4 years, in the absence of any surgical intervention.

**Discussion/conclusion:**

LHEP may represent a specialized form of degenerative epiretinal membrane associated with Muller cell activation. Spontaneous degenerative LMH closure may rarely occur with these lesion types, in the absence of surgical intervention, possibly due to Muller cell proliferation preceded by PVD.

## Introduction

Lamellar macular hole (LMH) with epiretinal proliferation or LHEP is a unique form of degeneration associated with secondary Muller Cell proliferation [1]. These ERM variants are not as amenable to a peeling procedure because of the friable Muller Cell component, the stable natural history, and the unpredictable surgical outcomes, and therefore the surgical option is often deferred [2]. Goveto et al. recently classified LMH into two subtypes: Tractional ERMs associated with outer macular schisis and a degenerative subtype consistent with LHEP [3].

In this report, an interesting case of LHEP is presented in which the lamellar macular hole spontaneously closed without surgical intervention. We present the sequential tracked OCT findings of this outcome and discuss the mechanism of closure which may relate to Muller cell activation preceded by posterior vitreous detachment (PVD).

## Case report

A 54-year-old female type 2 diabetic patient presented with a history of progressive vision loss of the left eye (OS). The right eye was legally blind due to end stage proliferative diabetic retinopathy (PDR).

On examination, best corrected visual acuity (VA) was hand motions in the right eye and 20/200 in the left eye. Ophthalmoscopic retinal examination OD was not possible due to a prepupillary fibrotic membrane. Retinal examination OS showed panretinal photocoagulation scars and evidence of regressed fibrotic PDR.

**Fig. 1:**
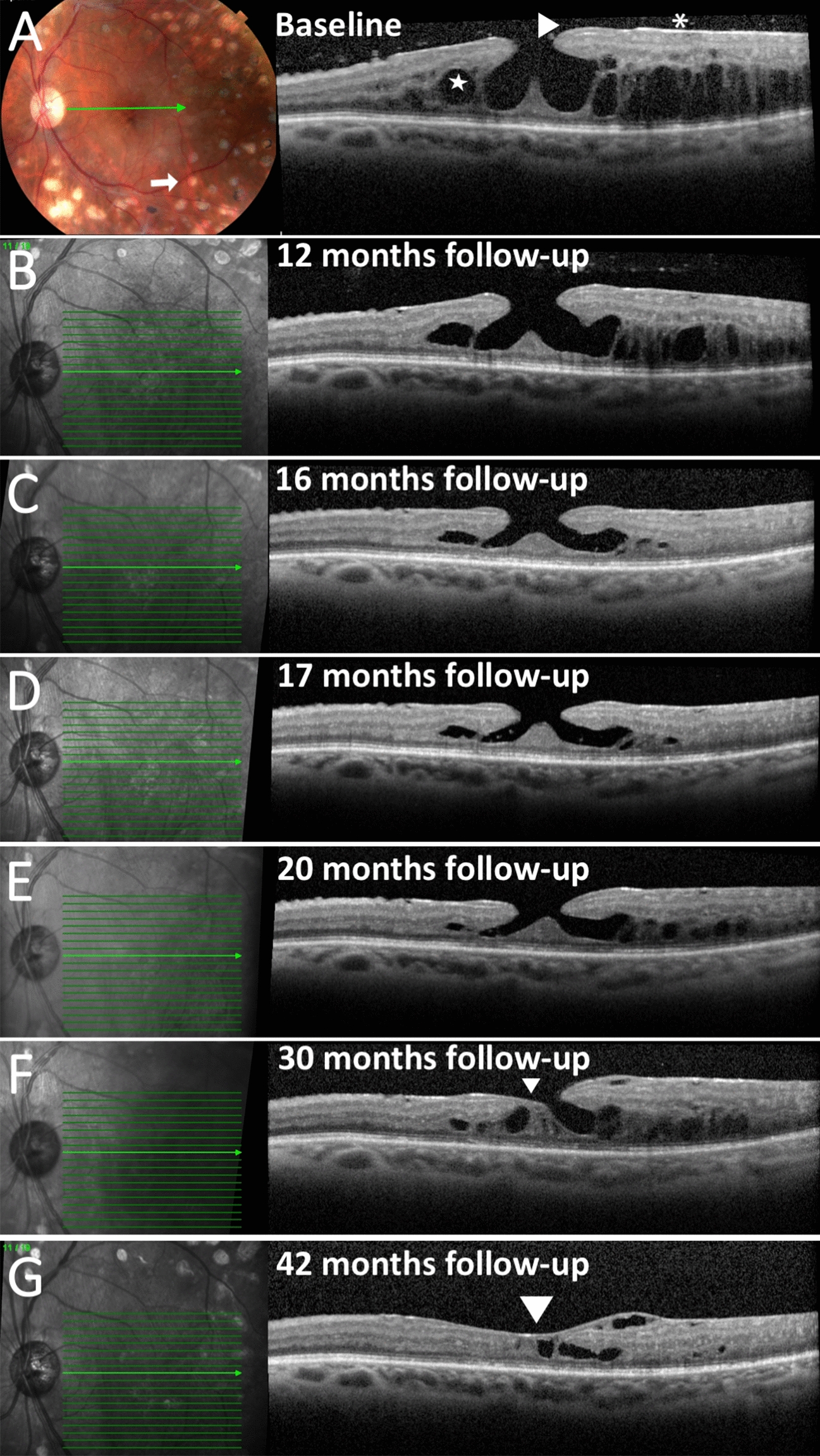
**A** Left. Fundus photography shows multiple panretinal photocoagulation laser scars consistent with regressed proliferative diabetic retinopathy. Right, SD-OCT B-scan illustrates cysts in the outer nuclear layer (star), lamellar hole with epiretinal proliferation (LHEP) (arrow-heads), and epiretinal proliferation (asterisk). Note the grade 0 posterior vitreous detachment. **B** Left. Registered near infrared reflectance with level of OCT section Right. **B**–**E** A partial grade 3 PVD is noted. While LHEP is unchanged, CME is improved. **E**–**G** Progressive closure of the LMH (arrowheads) and resolution of the CME is illustrated with sequentially tracked SD-OCT B scans. Complete grade 4 PVD is noted

Spectral domain – optical coherence tomography (SD-OCT) OS illustrated a lamellar macular hole with epiretinal proliferation associated with cystic degeneration predominantly located in the outer nuclear layer (Fig. 1A). A grade 0 PVD [4, 5] was also noted (Fig. 1A). The patient was treated with intravitreal bevacizumab injection (IVB) although the etiology of CME may have been related to traction associated with the ERM given the predominantly outer retinal location of the fluid.

SD-OCT 1 year later illustrated persistent LHEP, improvement of the CME and a partial grade 3 PVD (Fig. 1B). Sequentially tracked SD-OCT, obtained over the following 42 months, remarkably illustrated progressive closure of the LMH and near resolution of the CME associated with a complete PVD (Grade 4 PVD) (Fig. 1C–G). While the patient did receive 4 additional bevacizumab injections and focal macular laser, resolution of the CME may have been related to release of the PVD and closure of the lamellar macular hole. The final VA remained stable 20/200 OS.

## Discussion

Degenerative epiretinal membranes (ERM) or lamellar macular holes (LMH) with epiretinal proliferation (LHEP) represent a distinct subset of ERM easily differentiated from tractional ERMs [6]. Although LHEP was first described as a result of complications of PVD, surgical and pathological studies have clearly demonstrated Muller cell proliferation associated with these lesions that may result from more degenerative related mechanisms [1]. LHEP has been associated with other degenerative disorders and not solely the result of PVD [7–9].

Muller cell activation and proliferation may lead to non-surgical closure of the LMH that was documented in this case report with sequentially tracked SD-OCT over 2 years [1]. Bringmann et al. described the spontaneous closure of a full thickness macular hole and indicated that Muller cell activation was the likely mechanism [8]. Others have described the spontaneous closure of LHEP but did not indicate if the sequential B scans were tracked [10]. In our case, complete PVD evolution may have also contributed to spontaneous closure of the LMH as a result of vitreoretinal traction release although these forms of LMH, i.e. LHEP, are not the result of tractional pathways.

Surgical management may be deferred in these cases due to the degenerative Muller cell component and the stable natural history.

## Conclusion

LHEP may represent a specialized form of degenerative lamellar macular hole associated with Muller cell proliferation. Non surgical closure may rarely occur with these lesion types possibly due to Muller cell activation preceded by PVD.

## Data Availability

The data sets used and analyzed during the current study are available from the corresponding author on reasonable request.
